# Antimicrobial Prophylaxis in Clean Pediatric Surgical Procedures: A Necessity or Redundancy?

**DOI:** 10.7759/cureus.10701

**Published:** 2020-09-28

**Authors:** Muhammad Khalid Syed, Ahmad A Al Faqeeh, Alsayed Othman, Ahmed A Hussein, Hala Rajab, Salman Hussain, Syed Muhammad Jawad Zaidi, Sabahat K Syed, Saifullah Syed, Talal Almas

**Affiliations:** 1 Pediatric Surgery, King Fahad Hospital, Al Baha, SAU; 2 Pediatric Surgery, Al-Azhar University - Assuit Branch, Assuit, EGY; 3 Internal Medicine, Royal College of Surgeons in Ireland, Dublin, IRL; 4 Internal Medicine, Rawalpindi Medical University, Rawalpindi, PAK; 5 Internal Medicine, Jinnah Sindh Medical University, Karachi, PAK

**Keywords:** antibiotic prophylaxis, clean pediatric surgical procedures, surgical site infections

## Abstract

Background

The clinical utility of antimicrobial prophylaxis in clean pediatric surgical cases remains enigmatic. The present study aims to evaluate the prevalence of surgical site infections in instances where antibiotic prophylaxis is not employed prior to clean pediatric surgical procedures.

Methods

A retrospective cross-sectional study that included data of all pediatric clean surgical procedures from January 2018 till January 2020 was conducted. All children undergoing clean surgical procedures who did not receive antibiotics at least two weeks prior to the procedure were included in the study. The exclusion criteria included patients with congenital heart disease, ventriculoperitoneal shunt, nephrotic syndrome, immunodeficiency, and prior administration of antimicrobial prophylaxis. All patients were followed for two to four weeks for any signs of surgical site infections.

Results

Of the 178 patients included, 119 were male and 59 were female, with the mean age hovering at 8.19 ± 2.87 years. Orchidopexy and herniotomy were the most commonly performed surgical procedures, and were performed in 56 (31.46%) and 54 (30.33%) patients, respectively. Only one case of postoperative surgical site wound infection was reported, accounting for a prevalence rate of 0.56%.

Conclusion

In clean pediatric surgical procedures, the risk of surgical site infections is exceedingly low. The unnecessary use of antibiotics in children can cause deleterious adverse effects and promote antimicrobial resistance. In a carefully selected pediatric population, administration of antibiotic prophylaxis might confer no added benefit.

## Introduction

Surgical site infections (SSI) refer to infections that arise within 30 days after any surgical procedure [1]. SSIs represent a significant clinical burden, being the most common complication following any surgical procedure. They account for approximately 8% of deaths due to nosocomial infections [2]. In addition to their significant impact on morbidity and mortality, SSIs pose an increased financial burden on the healthcare system by resulting in longer hospital stays and increased cases of re-admission [3]. The use of appropriate evidence-based guidelines, including the timely and appropriate administration of prophylactic antibiotics, provides significant protection against SSIs [4,5]. This reduces the bacterial load necessary to cause infection at the surgical site [6]. The appropriate selection of antimicrobial agents as well as the employment of antibiotic prescription principles is therefore of immense importance in the reduction of antibiotic-resistant organisms [7,8]. It is noteworthy that prophylaxis implies the preventive use of antibiotics in the absence of infection or contamination [9].

In a majority of cases, the mainstay of SSI prevention is the appropriate selection and timely administration of a single dose of prophylactic antibiotics one hour prior to surgery [10]. While there has been tremendous data that substantiates this recommendation, this applies to specific procedures only. For instance, single-dose preoperative antibiotic therapy in elective abdominal surgery has been documented to reduce SSIs [11]. On the other hand, the role of antibiotic therapy in the reduction of SSIs, as it pertains to other surgical procedures including those for penetrating abdominal traumas, is more debatable [12]. For this reason, surgeons must be well-versed with the procedures for which antibiotic prophylaxis is mandated. In certain clean surgical procedures, where the rate of infection is significantly lower, a plethora of studies has demonstrated the ineffectiveness of prophylactic antibiotics in reducing SSIs [13,14]. Due to the paucity of controlled clinical trials in the pediatric population, the present study aims to evaluate the effectiveness of prophylactic antibiotics prior to clean pediatric surgical procedures.

## Materials and methods

This retrospective cross-sectional study was conducted in the department of pediatric surgery, King Fahad Hospital, Al Baha, Saudi Arabia. A total of 178 patients undergoing clean surgeries were included in the study. Children under 14 years of age who underwent elective clean surgical procedures as defined by the American Society of Anesthesiologists score I (ASA-I) and who were operated during 2018-2020 were included in the study. All patients were operated under aseptic conditions and total intravenous general anesthesia. Additionally, all patients were followed-up for two weeks. In suspected cases, the follow-up period was extended to four weeks in order to assess for features of surgical site infections such as erythema, purulent discharge, and wound dehiscence. Patients with comorbidities like congenital heart disease, ventriculoperitoneal shunt, nephrotic syndrome, and immune deficiency were excluded from the study. Moreover, patients who had a history of intravenous or oral antibiotic intake two weeks prior to the date of their procedure were also excluded from the study. The data was collected from the computed records and patients’ files. The study was approved by the ethical review board of King Fahad Hospital Al Baha, Saudi Arabia. The frequency and percentages of the various surgical procedures were tabulated. The prevalence of SSIs after each procedure was calculated. The data was eventually analyzed using the SPSS version 23 (IBM Corp., Armonk, NY).

## Results

The present study involved 178 patients undergoing elective clean surgical procedures, of which 119 were males and 59 were females. The female-to-male ratio was approximately 1:2. Table 1 delineates the breakdown of study participants based on gender.

**Table 1:** A breakdown of study participants with respect to gender.

Gender	Frequency (n)	Percentage (%)
Males	119	66.85%
Females	59	33.15%

The mean age of the study participants was 8.19 ± 2.87 years. Orchidopexy was the most common clean surgical procedure performed (56 out of 178 patients), followed by herniotomies (54 patients), and circumcision in 38 patients. Table 2 elucidates the various procedures performed and their respective frequencies.

**Table 2:** A depiction of the type of surgeries performed on patients. PPV: Patent processus vaginalis

Type of Surgery	Frequency (n)	Percentages (%)
Orchidopexy	56	31.46%
Herniotomies	54	30.33%
Circumcision	38	21.35%
Excision of superficial lumps	12	6.75%
Epigastric hernia repair	8	4.49%
Umbilical hernia repair	6	3.37%
PPV ligation for hydrocele	4	2.25%
Total	178	100%

Pertinently, surgical site infection (SSI) was noted in only one case of orchidopexy. One case with SSI out of 178 observed procedures accounts for only 0.56% of SSIs without the use of prophylactic antibiotics in clean pediatric surgical procedures, alluding to a very meagre percentage.

## Discussion

The effectiveness of the administration of prophylactic antibiotics is well established for certain surgical procedures, leading to a reduction in SSI rates, duration of hospital stay, as well as postoperative morbidity [14]. However, inappropriate administration of antibiotics, whether by the employment of a poor antibiotic regimen or the excessive use of antibiotics postoperatively, is a major reason for the emergence and spread of multi-drug resistant organisms in both hospital and community settings [8,15]. Kreisel et al. examined the relationship between prophylactic antibiotic therapy and the development of clostridium difficile toxin positivity and reported a five-fold greater risk of positivity with inappropriate prophylaxis [16]. Antibiotics are also the most common class of medications implicated in drug allergy and anaphylaxis [17].

Clean surgical wounds are defined as uninfected operative wounds in which no inflammation is encountered [18]. Surgical procedures resulting in clean surgical wounds typically manifest lower rates of infection. Studies have demonstrated no statistically significant reduction in rates of infection with the use of prophylactic antibiotics [5,19]. There is a considerable lack of literature in the pediatric population regarding the use of antibiotic prophylaxis in clean surgical procedures. Pertinently, there are no formal guidelines in pediatric urologic procedures that dictate the use of perioperative antibiotics [20]. Despite that, a study reported a very low postoperative wound infection rate of 0.8% across all surgical wound classifications. Furthermore, no notable differences were perceived in SSI rates with or without prophylactic antibiotic administration in pediatric patients undergoing clean surgical urological procedures [20].

Various studies involving pediatric surgery patients receiving prophylactic antibiotics have reported an increased risk of adverse events in comparison to those who did not receive prophylactic antibiotics. This included an increase in clostridium difficile infections, as well as an increased risk of allergic reactions [21,22]. Another study demonstrated no reduction in SSIs upon the administration of prophylactic antibiotics in patients undergoing orchidopexy, while also observing an increased risk of perioperative allergic reactions [13].

The present study demonstrates a very low rate of postoperative wound infection in clean surgical procedures without the administration of preoperative prophylactic antibiotics. These findings are in accordance with similar findings reported in various other studies [13,20-22]. Larger studies with greater sample sizes are needed to better elucidate the outcomes of SSIs in clean surgical procedures with no administration of prophylactic antibiotics, especially since there are no formal guidelines that can guide surgeons in making informed decisions.

## Conclusions

The rates of surgical site infections in clean pediatric surgical procedures remain exceedingly low. In this context, the administration of preoperative prophylactic antibiotics might be unwarranted and can contribute towards the pressing issue of mounting antimicrobial resistance. Nevertheless, the current literature remains largely bereft of such guidelines as they pertain to the pediatric population. There is thus an unmet need for curation of appropriate evidence-based guidelines in order to better inform the current debate.
